# Potential value of patient record review to assess and improve patient safety in general practice: A systematic review

**DOI:** 10.1080/13814788.2018.1491963

**Published:** 2018-08-16

**Authors:** Caoimhe Madden, Sinéad Lydon, Ciara Curran, Andrew W. Murphy, Paul O’Connor

**Affiliations:** a Department of General Practice, School of Medicine, National University of Ireland Galway, Galway, Ireland;; b HRB Primary Care Clinical Trials Network Ireland, National University of Ireland Galway, Galway, Ireland;; c School of Medicine, National University of Ireland Galway, Galway, Ireland

**Keywords:** Primary care, medical error, patient safety, record review, systematic review

## Abstract

**Background:** There is limited research, and guidance, on how to address safety in general practice proactively.

**Objectives:** This review aimed to synthesize the literature describing the use of patient record review (PRR) to measure and improve patient safety in primary care. The PRR methodologies utilized and the resulting outcomes were examined.

**Methods:** Searches were conducted using Medline, Embase, CINAHL and PsycINFO in February 2017. Reference lists of included studies and existing review papers were also screened. English language, peer-reviewed studies that utilized PRR to identify patient safety incidents (PSIs) occurring in general practice were included. Two researchers independently extracted data from articles and applied the Quality Assessment Tool for Studies with Diverse Designs.

**Results:** A total of 3265 studies were screened, with 15 included. Trigger tools were the most frequent method used for the PRRs (*n* = 6). The mean number of safety incidents per 100 records was 12.6. Within studies, a mean of 30.6% of incidents were associated with severe harm (range 8.6–50%), and a mean of 55.6% of incidents was considered preventable (range 32.7–93.5%). The most commonly identified types of PSIs related to medication and prescribing, diagnosis, communication and treatment. Three studies reported on improvement actions taken after the PRRs.

**Conclusion:** This review suggests that PRR may be a promising means of proactively identifying patient safety incidents and informing improvements.

KEY MESSAGES

- Patient record review offers the opportunity to identify instances of harm to a patient and to undertake quality improvement to improve patient safety.
- Despite the potential of patient record review to improve patient safety, there is a need for further research to ensure validity and reliability of the approach.

## Introduction

Despite increased interest in researching patient safety [1], general practice has received little attention in this regard due to a perception that it is relatively low-risk [2,3]. Nevertheless, research suggests that 2–3% of general practice consultations may result in adverse events [4], which is concerning given the high volume of patient contacts in these settings [5]. Such errors may be potentially preventable in 45–76% of cases [6], with serious harm occurring to 6–7% of patients [7]. These data emphasize the importance of investigating patient safety incidents (PSIs; defined by the World Health Organization [8] as ‘an unintended event during the care process that resulted, could have resulted, or still might result, in unnecessary harm to a patient’).

General practitioners have described difficulties in understanding how best to measure and improve patient safety in their practices [9]. Although a range of safety measurement systems have been identified [10], commonly used ‘reactive’ approaches to safety improvement (e.g. incident reporting systems) typically commence in response to a specific case of severe harm, which can have negative repercussions for the physicians involved [9], and questionable validity in terms of preventing future harm [11,12]. Therefore, there is a need for valid, reliable, feasible and acceptable methodologies to proactively monitor safety by identifying indicators of potential PSIs [1], allowing for constructive, practice-based quality improvement to be undertaken.

Conducting patient record reviews (PRRs) is a proactive safety measurement approach, whereby patient records are screened by trained clinicians to ascertain whether or not a patient has experienced a PSI [13], and information is extracted about the nature of the incident (e.g. cause, severity, and preventability [14]). PRRs allow corrective, systematic improvements to be taken, which may help to prevent the patient from future harm [15]. PRRs have been widely used within hospital settings [16,17] and have been identified as a promising measure of safety in general practice [12].

This review is different from systematic reviews previously conducted in this area, as it focuses on the value of PRR specifically for the proactive assessment of PSIs. Others have provided an overview of commonly used patient safety measurement tools [12], applied specific PRR methods such as trigger tools [18], or measured the validity and reliability of PRR [11].

Our aim was to provide an overview of the literature describing the use of PRR to measure and improve safety in general practice by delineating the various PRR methodologies and examining the characteristics of PSIs identified.

## Methods

### Search strategy

This review is reported in accordance with the PRISMA guidelines [19]. Systematic searches were conducted within four electronic databases in February 2017: Medline, Embase, CINAHL, and PsycINFO. The search protocol (see online supplementary material 1 for a sample search strategy) included Medical Subject Headings (MeSH) search terms along with other keywords. No limits were placed on publication year.

The reference lists of all included studies were manually screened, along with the bibliographies of the first and last author of each study, to identify additional relevant studies. The reference lists of three recent reviews pertaining to patient safety in primary care [4,10,12] were examined.

### Eligibility criteria

To be included, studies had to: be published in an English language, peer-reviewed journal; report original research and; describe the use of manual and/or automated PRR as a means of identifying PSIs either retrospectively and/or prospectively within general practice [20].

Studies were excluded due to: the description of PRR focused solely on those with a single medical condition or those prescribed a specific medication; the use of PRRs to evaluate one particular primary care process or function alone (e.g. prescribing or diagnosis only); the use of PRRs in a hospital setting only or a primary care setting other than general practice, or; PRR in an ambulatory care setting that did not provide primary care services.

### Study selection

Titles and abstracts were screened to assess suitability for inclusion. If these provided insufficient information to determine inclusion or exclusion then the full-text of the paper was examined.

### Data extraction and synthesis

A structured tool was used to extract information on study characteristics including PRR method, individual responsible for conducting PRR, inter-rater reliability, number of records reviewed, patient sample, and time taken to conduct the PRR. Specific outcome data were extracted including the rate of PSIs per 100 records, types of PSIs and their rate per 100 records, severity of PSIs, preventability of PSIs, and data relating to any improvement actions taken. Panesar and colleagues’ guidance on computing the number of incidents per 100 records reviewed was used to calculate outcome data [4]. Two authors conducted the data extraction independently and disagreements were resolved through discussion [21].

### Methodological quality assessment

Included studies were critically appraised using the Quality Assessment Tool for Studies with Diverse Designs (QATSDD) [22]. This instrument allows for the methodological assessment of studies using qualitative, quantitative, and mixed methods research designs. The QATSDD has been previously used in other systematic reviews, with high levels of agreement reported [12,23]. Scores on this measure can range from 0–48, with higher scores indicative of methodological rigour. Two reviewers completed the quality assessment and disagreements were resolved through discussion.

## Results

**Figure 1.**
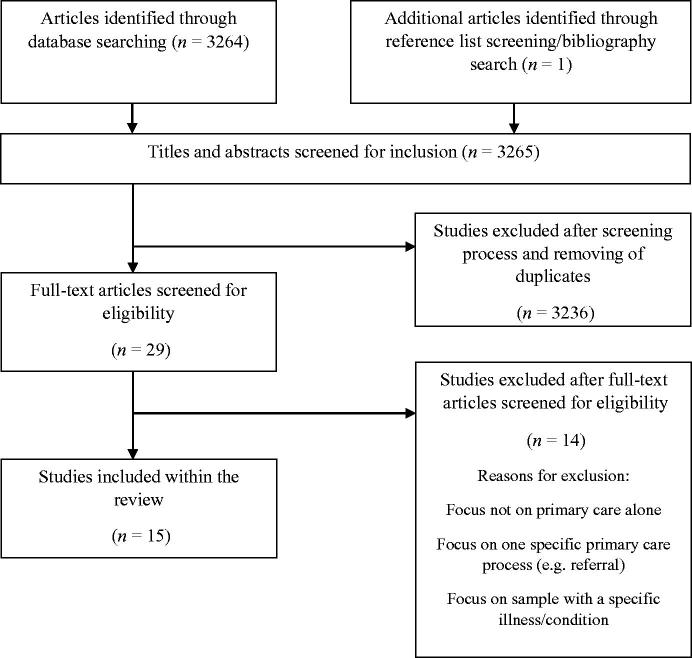
Identification of studies for review.

As shown in Figure 1, the electronic searches returned over 3200 papers, of which 15 studies were included. One additional study was identified through reference list and bibliography screening [24].

### Study characteristics

Fourteen studies were quantitative and one was qualitative [24]. Studies were published between 2003 and 2017. As shown in Table 1, studies were most frequently conducted in Europe (60%), followed by North America (20%), and Asia (13.3%).

**Table 1.** Characteristics of the 15 included studies, which assess the use of patient record review for detecting patient safety incidents in a primary care setting.

Characteristics	References	Number of studies (%)
**Study Location**^a^		
Europe	[15,24–31]	9 (60)
North America	[32–34]	3 (20)
Asia	[35,36]	2 (13.3)
South America	[34]	1 (6.6)
New Zealand	[37]	1 (6.6)
**Chart review method**		
Trigger tool/criteria		6 (40)
Trigger review method (10 triggers)	[15,24,25]	3 (20)
36 trigger criteria	[37]	1 (6.7)
9 trigger criteria	[26]	1 (6.7)
23 trigger criteria	[32]	1 (6.7)
Record review using error definition		5 (33.3)
WHO definition	[27–29]	3 (20)
Diagnostic/documentation/management definition	[35,36]	2 (13.3)
Clinical judgement		2 (13.3)
Physician panel judgement	[30]	1 (6.7)
Individual clinician judgement	[31]	1 (6.7)
Record review following patient report of errors		2 (13.3)
Patient interview	[34]	1 (6.7)
Patient survey	[33]	1 (6.7)
**Reviewer**^a^		
Physician	[15,24,25,27–30,32–37]	13 (86.7)
Practice nurse	[24,25,32,33,37]	5 (33.3)
Unidentified researcher	[31,34]	2 (13.3)
Trainee GP/medical students	[26,30]	2 (13.3)
Administrator	[15]	1 (6.7)
Pharmacist	[37]	1 (6.7)
**Patient sample**^a^		
Random sample	[24,25,27–29,31–33,35–37]	11 (73.3)
High-risk patient group	[15,24,26]	3 (20)
Random sample with specific criteria	[34,37]	2 (13.3)
Consecutive sampling	[30]	1 (6.7)
Deceased	[29]	1 (6.7)

a Figures do not total to 15 as some studies fit within more than one of the categories.

### Chart review method

Detailed descriptions of each study are presented in online supplementary material 2. As seen in Table 1, the use of a trigger tool to guide the screening process was the most frequent approach (40% of studies) [15,24–26,32,37], and involves searching records for the presence of predefined ‘triggers’, i.e. clinical prompts that may indicate the existence of PSIs [24]. The use of an error definition was the next most common (33.3%) [27–29,35,36], whereby a standardized definition (e.g. ‘an unintended event during the care process that resulted, could have resulted or still might result in harm to the patient’ [27]) was applied. Reliance on clinical judgement [30,31] (e.g. discussion by a panel of physicians) and PRR following patient-report of errors [33,34] (e.g. interview) were less frequently employed.

### Number of records

Two studies did not provide data on the number of records reviewed [15,34]. Across the remaining studies, the mean number of records reviewed was 1589.33 (SD = 3312.26; range: 28–13 351).

### Reviewer

Physicians most frequently conducted the PRRs (86.7% of studies). Nurses (33.3%) and unspecified researchers (13.3%) also served as reviewers. ‘Other’ reviewers included trainee GPs/medical students, administrators and pharmacists (13.3%, 6.7% and 6.7%, respectively).

### Patient sample

Random samples of records were screened in 73.3% of studies [24,25,27–29,31–33,35–37], whilst fewer studies (20%) selected a high-risk patient sample (e.g. patients >75 years of age [26], patients with a heart failure diagnosis [15]). ‘Other’ patient samples (e.g. deceased patients) were evaluated less frequently. Three studies [24,29,37] reviewed records from multiple sample types.

### Interrater agreement

The agreement between reviewers was reported in only six studies [25,27,30,32,34,37] (40%). Findings were variable, ranging from a ‘high level of agreement’ to ‘relatively low correlation’ [25,30,37]. Of those reporting interrater agreement, only two studies reported Kappa values [27,34], which ranged from substantial (κ = 0.63) [27], to almost perfect agreement (κ = 0.83) [34]. Further detail is provided in supplementary material 2.

### Time taken

Only three studies reported the time taken to perform the PRR [24–26]. The mean time taken to review one patient record was 5.33 min (SD = 1.91 min; range: 3.2–6.9 min).

### Quality of included studies

The mean QATSDD score was 19.67 (SD = 5.02; range =10–30) out of 48. Quality scores for individual studies are presented in Table 2. Studies generally performed well on items relating to the description of the aims/objectives, research setting, data collection procedure, and the fit between the research question and analysis. However, studies typically performed poorly on items relating to consideration of sample size and consideration of the measurement tools’ psychometric properties.

**Table 2.** Patient record review method and patient safety incident characteristics of the 15 studies assessing the use of patient chart review for detecting patient safety incidents in a primary care setting.

Method	Study	Quality score	Number of patient safety incidents per 100 records	Types of errors per 100 records^a^	
Trigger tool	Sears et al. [32]	30	14.2	Medication error = 2.3	
	DeWet et al. [24]	25	14.1	Medication/prescribing errors = 4.9 Diagnosis errors = 0.3	Communication errors= 0.8
	DeWet and Bowie [25]	24	12.8	Medication errors = 7.6	
	Eggleton and Dovey [37]	22	26.5	Medication errors = 26.5	
	McKay et al. [26]	22	15.4	Data not provided	
	Bowie et al. [15]	10	UTD	UTD	
Error definition	Gaal et al. [27]	19	21.1	Treatment errors = 3.1 Communication = 2.6 Diagnosis = 2.1	
	Khoo et al. [35]	19	UTD	Documentation errors = 98 Medication errors = 53.2	Diagnostic errors = 3.6
	Khoo et al. [36]	19	UTD	Intervention group (pre-intervention) Diagnostic error: 4.1 Medication errors: 43.2	Control group (pre-intervention) Diagnostic error: 3.4 Medication errors: 39
				Intervention group (post-intervention) Diagnostic error: 2.5 Medication errors: 25.2	Control group (post-intervention) Diagnostic error: 0.9 Medication errors: 36.7
	Martijn et al. [28]	17	UTD	Data not provided	
	Wetzels et al. [29]	14	Living patients: 7.3 Deceased patients: 14.8	Living patients Diagnosis errors: 0.7 Treatment errors: 2.7 Communication errors: 2.7	Deceased patients Diagnostic errors: 3.7 Communication errors: 7.4
Clinical judgement	Smits et al. [30]	21	2.4	Treatment errors = 1.3	Diagnostic errors = 0.5
	Wetzels et al. [31]	13	7.3	Therapeutic errors: 2.7 Communication errors: 2	Diagnostic errors: 0.7
PRR following patient report	Montserrat-Capella et al. [34]	22	UTD	UTD	
	Solberg et al. [33]	18	2.3 (‘real clinician errors’)	UTD	

*Note*: UTD: unable to determine.

a The most commonly identified types of Patient Safety Incidents are presented here. See Supplementary Material 2 for detail on other types of errors.

### Outcome data

#### Rate and types of PSIs

Table 2 presents data on the number and types of PSIs detected per 100 records per study. Five studies did not provide the data necessary for these calculations [15,28,34–36]. The mean number of PSIs per 100 records was 12.6 (SD = 7.21; range: 2.3–26.5). The most commonly identified types of PSIs related to medication and prescribing, diagnosis, communication and treatment. Online supplementary materials 2 presents data relating to other types of errors.

### Severity and preventability of PSIs

**Figure 2.**
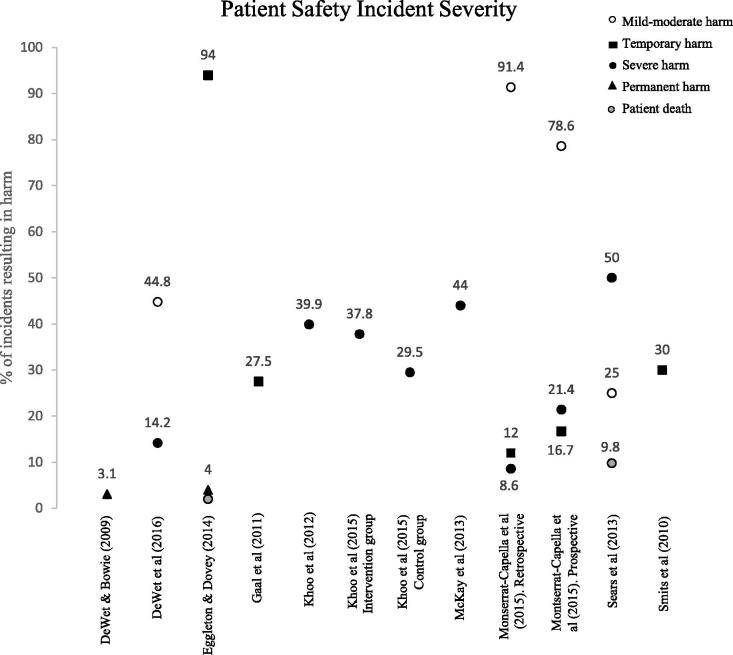
Severity of identified patient safety incidents per study.

Figure 2 provides an overview of the degree of harm resulting from the PSIs across included studies. Studies used similar rating scales to classify the harm resulting from PSIs, with severity categories ranging from ‘mild-moderate harm,’ ‘temporary harm,’ to ‘severe harm,’ ‘permanent harm’ and ‘patient death’ depending on the specific rating scale used. Four studies did not report severity of harm, and one study rated harm as ‘likely/unlikely’—these are excluded from Figure 2 [15,28,29,31,33].

**Figure 3.**
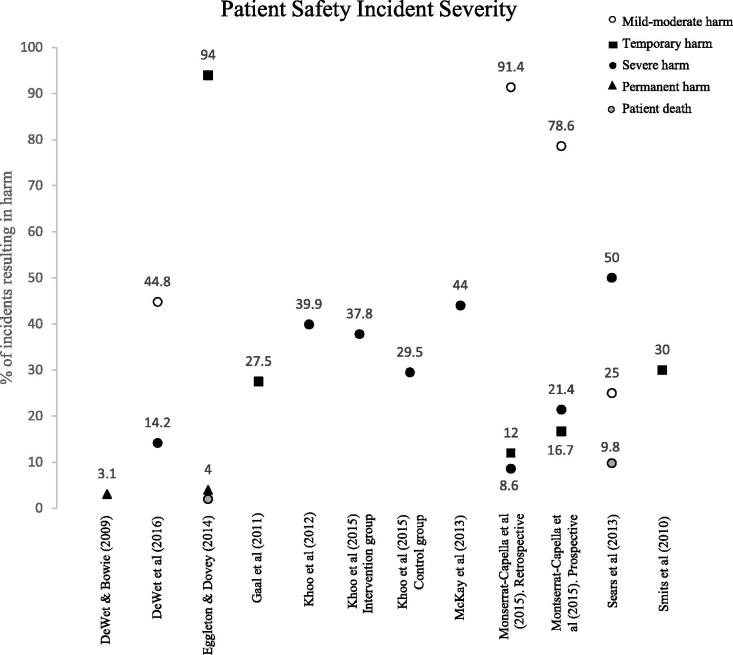
Proportion of patient safety incidents rated as preventable per study.

Seven studies reported the percentage of the total number of PSIs that were deemed by the expert reviewers to have been avoidable [24–26,32,34–36]. As shown in Figure 3, a mean of 55.6% of PSIs were considered preventable (SD = 19.6; range: 32.7–93.5).

### Improvement actions

Three studies reported on actions taken subsequent to the PRRs [15,24,26]. The most common actions included making a specific improvement, feedback to colleagues, clinical audit, and protocol updates (see online supplementary material 2).

## Discussion

### Main Findings

This review of 15 studies revealed a PRR approach to be a feasible and useful means of measuring, and potentially improving, safety in general practice. However, variation in the levels of harm, severity and preventability resulting from PSIs were observed.

### Interpretation in relation to existing literature

The use of a trigger tool is gaining recognition as a feasible and acceptable approach for identifying PSIs [15,24,26,38]. Studies using trigger tool methodologies tended to detect higher incidences of PSIs (M = 16.6) and had higher quality scores (M = 22.2) as compared to alternate methodologies such as the use of error definitions (M PSIs per 100 records = 14.4; M quality score = 17.6), clinical judgement (M PSIs per 100 records = 4.9; M quality score = 17) and patient report (M PSIs per 100 records = 2.3; M quality score = 20), suggesting greater empirical support for the use of a trigger tool approach. Comparatively, the low-quality scores of, and low number of PSIs identified within, studies using clinical judgement or error definitions may suggest that these PRR methodologies require further research and refinement. Identified limitations include evidence of differing understandings of medical error among practitioners and critique of interview data for being over-reliant on recall [3,38], expensive, and time-consuming [27]. However, patient and/or physician interviews may be useful for gathering detail regarding contributory factors for PSIs [27,30].

In the current review, a mean of 12.6 errors per 100 records were identified across the studies. A previous review looking at the use of record review and prescription review in the context of safety measurement reported approximately 2–3 PSIs per 100 consultations/records (range: <1 to 24) [4], a notable discrepancy. It has been suggested that PSIs in general practice are often unreported [39], and PRR can capture these unreported data. This finding may explain the higher rates of errors in our review, which is focused solely on PRR methodologies as compared to a previous study of safety in primary care [4]. The most common types of errors (medication/prescribing, diagnosis, communication, and treatment) identified are in agreement with the findings of other research [7], and data showing that diagnostic and medication errors are the source of common general practice malpractice claims [40].

### Strengths and limitations

A thorough search strategy was employed, there was no specified publication year range, and reference list checks of related reviews were performed. The resultant data was extracted independently by two researchers to ensure maximum accuracy.

However, there are limitations to our methodology. First, the exclusion of studies describing measurement tools focused on assessing specific safety issues within primary care (e.g. prescribing errors) may be disputed. However, the current review aimed to provide a broader perspective on improving overall safety in general practice rather than targeting specific areas [9]. Second, the analysis and figures described in this review did not consider possible contributory factors that may have impacted them (e.g. high-risk patient groups, location) as it was beyond the scope of this review. Finally, limiting the searches to English language and the exclusion of grey literature may have resulted in PRR data being omitted (e.g. audits presented in general practice magazines) and a possible over-estimation of intervention effectiveness [41]. However, there is limited guidance on the methodological reproducibility of grey literature searches [42].

### Implications for research and practice

#### Refined methodologies

Some recommendations can be made concerning the use of a PRR approach in general practice. First, it is apparent that PRR can yield valuable data that may contribute to safety and quality improvement in general practice. However, these methodologies have been employed in a relatively small number of studies and further research is necessary to refine their methodologies for maximal efficiency and effectiveness. For example, criticisms of PRRs may include the risk of hindsight bias and an over-dependency on data quality (i.e., PRRs are completely reliant on the accuracy [43], completeness and legibility of patient records) [14]. Therefore, differing methodologies can contribute to varying estimates of PSIs [44]. There is a need to refine and standardize the methods used in PRR to improve consistency and validity and facilitate ease of comparison across studies and between different practices.

Poor levels of agreement between reviewers are often reported—if reported at all [45]. The provision of adequate training and educational materials may be one approach to improving the reliability of reviewers [26].

### Predictors of harm

There was substantial variation in the levels of harm observed across studies (range = 2.3–26.5 PSIs per 100 records). Analysis of harm could allow for the identification of predictors such as specific characteristics of practices or patients. Future research should provide a deeper insight into the contributory factors surrounding PSIs and potential means of averting them.

### Triangulation

Triangulation of multiple measures of patient safety has been recommended by some researchers [4,29], and it has been suggested that patient safety cannot be encapsulated using one standalone methodology [1]. Recent systematic reviews examining patient safety measurement tools have provided useful information on the diversity of information provided by various methods (e.g. event reporting systems and mortality reviews typically focus on past harm) [10,12]. Staff survey techniques are a commonly utilized measure of patient safety [12], although discrepancies in safety climate reporting have been previously reported depending on managerial position [46]. Methods depending on patient report are resource intensive and over-dependent on recall but can give additional insight regarding the context surrounding the occurrence of PSIs [12]. Considering the varying nature of incidents detected according to method, there is strong rationale for combining more than one method of studying patient safety.

Future research should evaluate the use of PRR in conjunction with other measures of patient safety and compare the resulting outcomes; for example, data on time taken to conduct measurement (reported in three studies [24–26]); this is of paramount importance, as it has been established that time pressures are a significant barrier in carrying out safety measurement in general practice [9,44,47]. Such exercises would allow for the identification of measures that may be likely to over- or under-estimate harm and would inform practitioners about the most feasible and useful safety measurement methodologies.

## Conclusion

It has been established that a lack of available tools to measure patient safety in general practice limits the ability to prevent PSIs and improve quality of patient care. Although a relatively small body of research has described the use of PRRs, the current review suggests that they may be a promising means of identifying PSIs and allowing practitioners to take proactive action to improve patient care. Although data primarily supports the use of a trigger tool to guide PRRs, there is a need for future research to refine methodologies and ensure adequate training of practitioners to conduct PRR and to action the resulting data.

## Supplementary Material

Supplementary Material 2

Supplementary Material 1
