# Supplementary material for: Potential value of patient record review to assess and improve patient safety in general practice: A systematic review
Source: Eur J Gen Pract. 2018 Aug 16;24(1):192–201. doi: 10.1080/13814788.2018.1491963 (PMC6104614; doi:10.1080/13814788.2018.1491963)
Supplement: Supplementary Material 2 [file IGEN_A_1491963_SM2659.docx]

**Supplementary Digital Content 2: Detailed account of Patient Record Review method and resulting outcomes for each of the included study.**

| **Study** | **Country** | **Chart review type** | **Chart review method** | **Audit conductor** | **Inter-rater reliability** | **Number of records** | **Clinical sample** | **Types of errors per 100 records** | **Intended actions** |
| --- | --- | --- | --- | --- | --- | --- | --- | --- | --- |
| Bowie et al. [15] | Scotland | Trigger review method | Reviewers used 10 ‘triggers’ to help them identify patient safety incidents:  1. Timing of consultation  2. Place of consultation  3. Frequency of consultation  4. Changes to medication  5. Adverse drug events/allergies  6. New clinical read code  7. Abnormal blood results  8. Out-of-hours and/or A&E  9. Hospital admission/ discharge  10. >1 outpatient appointments in last year | At least two clinicians and an administrator were trained within each participating practice | Not recorded | A minimum of 20 records were reviewed within “around” 40 participating practices on a quarterly basis | Patients with a diagnosis of heart failure or taking high-risk medication (methotrexate or warfarin) | Unable to determine | Across the nine incidents, actions taken were:  Making a specific improvement (45%); Feedback to colleagues (18%); clinical audit (18%); Protocol update (18%) |
| DeWet & Bowie [25] | Scotland | Trigger review method | Reviewers used 10 ‘triggers’ to help them identify patient safety incidents:  1. Timing of consultation  2. Place of consultation  3. Frequency of consultation  4. Changes to medication  5. Adverse drug events/allergies  6. New clinical read code  7. Abnormal blood results  8. Out-of-hours and/or A&E  9. Hospital admission/ discharge  10. >1 outpatient appointments in last year | Five GPs and two practice nurses were trained to conduct the audits | Two auditors independently reviewed all records. Auditors reported a “high level of agreement” | A random sample of 100 clinical records was selected from five participating GP practices (n = 500) | Any patient registered with the practice for over 12 months. | Medication errors= 7.6  Administration errors=2.8  Procedural errors= 1.6 “Other” errors= 0.8 | Not recorded |
| DeWet et al. [24] | Scotland | Trigger review method | Reviewers used 10 ‘triggers’ to help them identify patient safety incidents:  1. Timing of consultation  2. Place of consultation  3. Frequency of consultation  4. Changes to medication  5. Adverse drug events/allergies  6. New clinical read code  7. Abnormal blood results  8. Out-of-hours and/or A&E  9. Hospital admission/ discharge  10. >1 outpatient appointments in last year | One or more clinicians (GP or practice nurses) trained per practice- 92.7% of reviews were conducted by GPs | Not recorded | 50 records screened annually in 318 general medical practices- 13,351 records reviewed during data collection for this study | First, a ‘high-risk’ patient group.  Then, ‘high-risk’ or random. | Medication/prescribing errors= 4.9  Monitoring errors= 2.4  Communication errors= 0.8  Diagnosis errors= 0.3  Healthcare-acquired infection= 0.1  Medical equipment errors= 0.1  Coding/record-keeping errors= 1.1 Investigations errors= 0.4 Unclear= 3.8 | 2,177 actions were planned as a result of the patient chart reviews; Feedback to colleagues was the most frequent action taken (41.2%), followed by add to appraisal documentation (18.1%), followed by significant event analysis (11.5%), make a specific improvement (10.1%), protocol update (8.7%), clinical audit (6.3%) and PDSA cycle (1.5%). 2.5% of actions were classified as “other”. |
| Eggleton & Dovey [26] | New Zealand | Trigger tool | Reviewers initially used 36 ‘triggers’ to help them identify patient safety incidents:  1. Adverse reaction recorded  2. Address of a residential facility  3. Home visit  4. >2 consults in a week  5. >12 consults per year  6. >3 consults with different GPs in a 3 month period  7. Predominant provider and nominated provider are different  8. No appointment and repeat prescription  9. No appointment and telephone prescription  10. Long-term medications and classifications are at variance  11. Diagnosis of cancer in the last 12 months  12. Cessation of medications  13. >6 medications prescribed  14. Change of medications  15. Reduction in medication dose  16. Hospital discharge- including emergency department and day stay  17. ED/A&M clinic after GP consult within 2 weeks  18. ED/A&M clinic within 2 weeks prior to GP consult  19. ED/A&M clinic after nurse consult within 2 weeks  20. ED/A&M clinic prior to nurse consult within 2 weeks  21. Hospital admission with no GP consult within 6 months  22. Attended outpatient clinic  23. INR (5+)  24. Histology  25. Abnormal gynaecology cytology  26. Lab results: eGFR <35 mL/min/1.73m2  27. Lab results: TSH <0.03 on thyroxine  28. Lab results: Carbamazepine (Tegretol) >40 umol/L  29. Digoxin (Lanoxin) >2 nmol/L  30. Phenytoin >80 umol/L  31. Theophylline >110 umol/L  Valproic acid >700 umol/L  32. Lithium >1.5 mmol/L  33. Short-term admission to residential aged care facility  35. Death  36. Medication list not complete  The refined ‘trigger’ tool consisted of 8 triggers:  (Developed as part of the study)  1. adverse drug reaction documented in the record  2. ≥2 consultations with a GP in the same practice in a week  3. Cessation of medication  4. Reduction in medication dose  5. ≥ 6 medications prescribed  6. Attending the emergency department or an after hours provider within 2 weeks of having seen a GP  7. eGFR<35  8. Death | Two teams reviewed records independently- one team consisted of a GP and a community pharmacist and the other team consisted of a GP and a practice nurse. | Two teams reviewed records independently; there was a “relatively low correlation” between decisions made by the two sets of reviewers | 170 patient charts were reviewed. | To be included in the review, patients had to have been registered with the practice for more than 12 months and to have had one GP visit within the year. 50% of records reviewed were from Maori patients. | Medication errors= 26.5 | Not recorded |
| Gaal et al. [29] | Netherlands | Record review using error definition | Reviewers used a definition of a patient safety incident (“an unintended event during the care process that resulted, could have resulted, or still might result in harm to the patient”) in order to identify patient safety incidents within patients’ charts. Reviewers only looked for incidents that could have been prevented. | Patient records were independently screened by two GPs. When a potential patient safety incident was identified, the record was reviewed by a third GP. | A sample of 50 records was reviewed by all three GPs; Agreement values ranged 82 and 86% for the presence of a patient safety incident. Classification of type of incident: k = 0.642. First given ECM code: k = 0.736. Severity of harm classification: k = 0.634. | 1,000 patient records from 20 general practices (50 patient records per practice) | Patients who had visited or contacted the practice over a 3 month period were randomly selected | Organisation errors= 11.6  Treatment errors= 3.1  Communication=2.6  Diagnosis=2.1  Prevention= 1.4 Triage= 0.3 | Not recorded |
| Khoo et al. [31] | Malaysia | Record review using error definition | Reviewers used the following definitions to facilitate the detection of patient safety incidents within patient charts:  “Diagnostic errors were deemed to have occurred when the history or physical examination did not match the problem or diagnosis stated in the medical records. Management errors were deemed to have occurred if there was an error in investigation, medication or in the decision making process. Documentation errors were deemed to have occurred when there were missing or inadequate documentation of history, examination, diagnosis in the medical records or problems of illegibility. Diagnosis and management errors were deemed inconclusive when the reviewers could not reach a conclusion due to illegibility, insufficient information or poor documentation” | Each record independently reviewed by 2 family medicine specialists | Each record independently reviewed by 2 family medicine specialists- level of agreement not reported | 1,753 patient records from 12 primary care clinics | Patient records were selected by systematic random sampling from each clinic | Documentation errors= 98  Medication errors= 53.2  Investigation errors= 21.7  Decision making errors= 14.5  Diagnostic errors=3.6 | Not recorded |
| Khoo et al. [32] | Malaysia | Record review using error definition | Reviewers used the following definitions to facilitate the detection of patient safety incidents within patient charts:  Diagnostic errors: Errors deemed to have occurred when the history or physical examination did not match the problem or diagnosis stated in the medical records.  Management errors: Errors deemed to have occurred if there was an error in investigation, medication, or in the decision making process.  Documentation errors: Errors deemed to have occurred when there were missing or inadequate documentation of history, examination, diagnosis in the medical records, or problems of illegibility.  Inconclusive diagnostic and management error: Errors deemed to have occurred when the reviewers could not reach a conclusion due to illegibility, insufficient information, or poor documentation. | Each record independently reviewed by 2 family medicine specialists | Reliability assessed by comparing reviewers’ judgements to that of the research team- level of agreement not reported | 12 primary care clinics (6 intervention group, 6 control) participated with 1,753 and 1,793 records reviewed pre- and post- intervention | Random sampling of outpatient paper records | Intervention group (Pre-Intervention) Documentation error: 98.3 Diagnostic error: 4.1 Investigation errors: 20.1 Medication errors: 43.2 Decision making errors: 12.4  Intervention group (Post-Intervention) Documentation error:76.1 Diagnostic error: 2.5 Investigation errors: 13.3 Medication errors: 25.2 Decision making errors: 8.7  Control group (Pre-Intervention) Documentation error: 97.4 Diagnostic error: 3.4 Investigation errors: 23.4 Medication errors: 39 Decision making errors: 17  Control group (post-intervention) Documentation error: 89.5 Diagnostic error: 0.9 Investigation errors: 12.7 Medication errors: 36.7 Decision making errors: 8 | Not recorded |
| Martijn et al. [30] | The Netherlands | Record review using error definition | Reviewers used the WHO’s definition of a patient safety incident (“an unintended event during the care process that resulted, could have resulted or still might result in harm to the patient) to guide their patient chart reviews. | Trained GPs | Not recorded | 50 records reviewed within each of 17 participating general practices (850 total) | Random sampling of patient records | Not recorded | Not recorded |
| McKay et al. [27] | Scotland | Trigger Review method | Reviewers used the following triggers to facilitate their identification of patient safety incidents:  1. ≥3 consultations  2. New ‘high priority’ code added  3. New allergy code  4. Repeat medication item discontinued  5. Out-of-hours/A&E attendance  6. Hospital admission  7. Hb< 10.0  8. eGFR reduction ≥5  9. Optional triggers | 21 GP trainees who had attended a 2 hour training workshop | Not recorded | 21 GP trainees reviewed a total of 520 patient records. | Patients with Ischaemic Heart Disease, Patients over 75 years of age, or patients from other ‘high-risk’ or relevant groups | Not recorded | A number of practical actions taken by trainees during the review process (mostly medication related) e.g. : reviewing prescribed items, amending dosages, arranging for monitoring through further blood tests.  The most common actions that were to be taken following the review were:  Feedback to colleagues (36.3% of incidents), discussion with educational supervisor (35% of incidents), addition to appraisal documentation (23.8% of incidents), significant event analysis (12.5%). Clinical audit (12.5%), make a specific improvement (10%), protocol update (8.8%), and PDSA cycle (3.8%) |
| Montserrat-Capella et al. [27] | Latin America (Mexico, Peru, Brazil and Colombia) | Patient Chart Review following Patient Report of Errors | Patients were interviewed about health issues they had experienced over the past 6 months in order to identify any patient safety incidents.  A patient safety incident was defined as “an event that results in unintended harm to the patient by an act of commission or omission rather than by the underlying disease or condition of the patient”  All patient safety incidents reported were subsequently investigated by reviewing the patients’ charts. | An Institutional Coordinator Team was created from physicians and interviewers within each participating health centres. This team reviewed patient records in which there was suggested to be a patient safety incident. | Adequate level of agreement (≥ 81.1%) between reviewers on patient safety incident occurrence, type of patient safety incident classification, impact of patient safety incident on patients, and judgement on preventability of patient safety incident.  Determination of whether an injury had occurred = 0.8111, determination of unexpected death = 0.772, determination of whether health care/self care/ informal caregiving contributed to injury = 0.791, overall determination of an AE = 0.828, determination of preventability = 0.714. | Not recorded | Randomly selected patients over 18 years of age. Patients were excluded if: they were pregnant, had been hospitalised in the past 30 days, were scheduled to be hospitalised in the following 42 days, were difficult to follow up with or unable to provide unreliable information, terminal patients under palliative care or who could only provide information through a legal guardian, patients assisted at surgical or invasive procedure services, patients participating in other clinical trials, and patients with pending medico-legal claims. | Unable to determine | Not recorded |
| Sears et al. [28] | Canada | Record review using trigger criteria | Patient chart reviews were a two stage process.  First, trained nurse reviewers used 23 screening criteria in order to identify patient charts that might have contained an undetected patient safety incident. These 23 screening criteria, or ‘triggers’, were:  1. unplanned admission to acute care hospital  2. Unplanned visit to hospital emergency department  3. Patient injury, harm, trauma or complication during community care access center (CCAC) admission  4. New problem/diagnosis noted during index CCAC admission  5. Recognised actual or potential environmental risks  6. Inappropriate/inaccurate CCAC or service provider assessment or patient  7. Acquired infection/sepsis  8. Other patient complications  9. Unplanned assessment/treatment by primary care provider  10. Unplanned admission to CCAC within the 6 months after discharge from index admission  11. Unplanned transfer/request for admission to long-term care facility  12. Development of neurological deficit not present on admission but present at the time of discharge from index admission  13. Unexpected death  14. Dissatisfaction with care documented in patient record and/ or evidence of complaint lodged  15. Unplanned admission to any hospital within the 6 months after discharge from index admission  16. Expected family/informal caregiver availability for patient assistance not realised  17. Adverse drug reaction  18. Adverse event reported by caregiver  19. Inappropriate discharge/inadequate discharge plan for index admission  20. Request for admission to CCAC within the 6 months after discharge from index admission  21. Cardiac/respiratory arrest  22. Documentation or correspondence indicating litigation  23. Any other undesirable outcomes not covered above.  Second, doctors reviewed charts that had one or more of the screening criteria in order to determine if they contained a patient safety incident. Three criteria were necessary to determine that a patient safety incident was present:  1. The presence of an injury to the patient, that  2. required at least the use of additional healthcare resources and  3. where the injury was assessed as resulting from the care received rather than from the underlying disease or condition. | Nurses initially reviewed charts for the presence of one or more of the screening criteria. Then, doctors reviewed charts that met one or more of the screening criteria. | Interrater reliability was assessed by comparing the results on 20% of the selected charts; there was substantial agreement between the raters | Of the 430 charts in the sample, 286 charts screened positive for one or more of the trigger criteria and were reviewed by a physician. | Stratified, random sample of discharged home care patients which includes patients with care requirements due to acute and chronic medical conditions, surgical recuperations, pre- and perinatal care, palliative care, disability/rehabilitation needs and trauma. | Falls with injury= 3.5  Medication error= 2.3  Pressure ulcer/skin breakdown= 1.6  General decline= 1.6  Delayed healing= 1.4 Infection= 1.2  CHF= 0.9 Catheter injury= 0.7  Bowel impaction/ obstruction=0.5 Bleed= 0.2 Dehydration= 0.2 | Not recorded |
| Smits et al. [34] | The Netherlands | Patient record review using clinical judgement | First, reviewers examined patient records to determine whether the healthcare the patient had received was potentially unsafe.  Second, the records of patients who had receive potentially unsafe healthcare were discussed by a panel of physicians to determine if a patient safety incident had occurred.  Third, the panel of physicians classified the incidents according to the type, causes, and consequences. | Phase one: An experienced GP and a final year medical student.  Phase two and three: panel of physicians. | 50 records were assessed by both reviewers in order; Agreement between the reviewers was high (98%). | 1,145 patient records from four GP cooperatives | Consecutive sampling used to identify the first 250 patients coming into contact with each cooperative. A contact included a GP visit, telephone advice from GP, or a home visit from a GP. | Treatment errors= 1.3  Triage errors= 0.8 Diagnosis errors= 0.5 | Not recorded |
| Solberg et al. [36] | USA | Patient Chart Review following Patient Report of Errors | A survey was posted to patients asking them about errors that had occurred during their care.  The Institute of Medicine’s definition of errors (“the failure of a planned action to be completed as intended or the use of a wrong plan to achieve an aim”) and safety (“freedom from accidental injury”) were used within this study.  A nurse reviewed all 1998 surveys that were returned and viewed patient records where there was consent to do so and an error had been reported. The nurse classified the type of complaint and whether the patient chart required review. Following this, the patient survey and the nurse’s form were sent to one of two experienced primary care physicians. Ambiguous cases were discussed among the three reviewers, patients were asked for further details where necessary, and a decision was made concerning whether a possible or probable medical error had occurred.  Possible or probable medical errors were reviewed by the department’s chair and a decision was made on whether the case should undergo formal committee peer review. | A nurse was first responsible for reviewing surveys and charts, followed by two experienced primary care physicians | Multiple raters (one nurse, two doctors) involved in process but agreement data not reported. | Patients reported 247 perceived PSIs and these were investigated via patient chart review | Patient names were selected randomly from adults or parents of children younger than 12 with an office visit to a medical group physician in the preceding two weeks. No more than eight patients were sampled per physician. | Unable to determine | Not recorded |
| Wetzels et al. [33] | Netherlands | Record review using error definition | Two forms of patient chart review were conducted:  First, a random sample of patient charts were reviewed.  Second, the charts of all deceased patients were reviewed.  For the purposes of both reviews. An adverse event was defined as “an unintentional event with actual or potential harm to the patient’s health status” | Two clinical researchers (both GPs) examined the random sample of patient charts  One GP examined the records of all of the deceased patients. | Not recorded | For the review of the random sample of patient charts:  30 medical records were reviewed for 5 different GPs (n=150)  For the review of the deceased patients’ charts, the charts of all patients who had died within a six month period were reviewed (n=28) | A random sample of patient charts (n=150) were reviewed.  The charts of all patients who died within a six month period (n=27) were reviewed. | Living patients Administration errors: 1.3 Diagnosis errors: 0.7 Treatment errors: 2.7 Communication errors: 2.7  Deceased patients Administration errors: 3.7 Diagnosis errors: 3.7 Communication errors: 7.4 | Not recorded |
| Wetzels et al. [35] | Netherlands | Patient record review using clinical judgement | Randomly selected patient records were examined by two clinical researchers independently for signs of adverse events. | Two clinical researchers | Not recorded | 150 medical records (30 per GP) | Randomly selected patients who had visited the GP office in the past five months. | Administration errors: 2  Therapeutic errors: 2.7  Communication errors: 2 Diagnostic errors: 0.7 | Not recorded |
