# Supplementary material for: Potential value of patient record review to assess and improve patient safety in general practice: A systematic review
Source: Eur J Gen Pract. 2018 Aug 16;24(1):192–201. doi: 10.1080/13814788.2018.1491963 (PMC6104614; doi:10.1080/13814788.2018.1491963)
Supplement: Supplementary Material 1 [file IGEN_A_1491963_SM2658.docx]

**Summary of Medline OVID Search Strategy**

1: Primary care.ti,ab.

2: Primary Health Care.ti,ab.

3: General Practice.ti,ab.

4: Family Practice.ti,ab.

5: Ambulatory care.ti,ab.

6: Community care.ti,ab.

7: 1 or 2 or 3 or 4 or 5 or 6

8: exp Organizational Culture/

9: exp Medical Error/

10: (safe* adj2 manag*).ti,ab.

11: (safe* adj2 culture*).ti,ab.

12: (safe* adj2 climate*).ti,ab.

13: (patient* adj2 safe*).ti,ab.

14: (patient* adj2 harm).ti,ab.

15: (safe* adj2 attitude*).ti,ab.

16: (safe*adj2 behav*).ti,ab.

17: (diagnos* adj2 error*).ti,ab.

18: (iatrogenic adj2 disease).ti,ab.

19: (adverse adj2 event*).ti,ab.

20: 8 or 9 or 10 or 11 or 12 or 13 or 14 or 15 or 16 or 17 or 18 or 19

21: 7 and 20

22: limit 19 to (English language and humans and (addresses or autobiography or bibliography or comment or congresses or dictionary or directory or duplicate publication or editorial or festschrift or in vitro or interactive tutorial or interview or lectures or legal cases or legislation or letter or news or newspaper article or overall or patient education handout or periodical index or portraits or two study or video-audio media or webcasts)).

*Note:* Exp= explode, ti= title, ab= abstract,
